# Novel Allelic Mutations in *Dw3* Gene That Affect the Height of Sorghum Plants

**DOI:** 10.3390/ijms252212000

**Published:** 2024-11-08

**Authors:** Ping Wang, Bingbing Liang, Zhengjun Li, Chunyu Wang, Lixia Zhang, Xiaochun Lu

**Affiliations:** 1 Institute of Plant Protection, Liaoning Academy of Agricultural Sciences, Shenyang 110161, China; pingw-556@163.com (P.W.); liangbb2121@163.com (B.L.); 2 Institute of Sorghum, Liaoning Academy of Agricultural Sciences, Shenyang 110161, China; 18608900201@163.com (Z.L.); wangchunyu88899@126.com (C.W.)

**Keywords:** BSA-seq, *Dw3* gene, novel mutations, sorghum

## Abstract

Breeding for dwarfing traits in sorghum is crucial. However, only three genes (*Dw1*–*Dw3*) that control plant height have been mapped. In this study, 634 sorghum cultivars were collected to investigate plant height and genotypes. Four were genotyped *Dw1DW2Dw3* (wild type) but with different plant heights, and they were selected to construct two populations and map new dwarf genes. Bulked segregant analysis with whole-genome resequencing of the two populations identified the candidate gene in one same genomic region—on chromosome 7. Then, it was narrowed down to a region containing nine genes. Amino acid and DNA sequence analysis of the parent and offspring plants revealed that two novel allelic mutations in the *Dw3* gene play a role in reducing the plant height—8R262 or 8R417, including 1 bp substitution and 2 bp deletions. Furthermore, we sequenced 19 cultivars that primarily exhibited a “one-dwarf” hybrid or wild-type and presumed another allelic mutation via the amino acid alignment of 8R019, 8R100, and 8R402, which was another one-base substitution. These results indicate that multiple types of allelic mutations in the *Dw3* gene should be considered when identified or applied.

## 1. Introduction

A variety of biomass energy sources can produce biofuels, including crops, energy crops, aquatic plants, and forestry waste [1,2]. Among them, Sorghum [*Sorghum bicolor* (L.) Moench.] is a staple food crop for half a billion people and an emerging biofuel crop [3]. Furthermore, sorghum is an important crop species for farmers in semiarid and arid regions. Because rising temperatures and reduced precipitation due to climate change make some areas unsuitable for maize and rice production, the importance of drought-tolerant crops like sorghum is likely to increase [4].

Breeding for dwarfing traits in sorghum is crucial because it enables mechanical harvesting, improves lodging resistance, and increases fertilizer use efficiency. Four major dwarfing genes, identified as *Dw1*–*Dw4*, control height by modifying the internode length [5]. In 2016, the gene corresponding to *Dw1* underwent map-based cloning using an F2 population and HIFs derived from Hegari and 80M. It was shown that the Dw1 protein could affect cell proliferation activity in the internodes [6,7]. As a result of mutations, *dw2*, located on chromosome 6, greatly reduces internode lengths [8,9]. Similarly, *Dw3*, located on chromosome 7, was finally shown to encode a transmembrane protein of the adenosine triphosphate-binding cassette (ABC) transporter superfamily involved in auxin efflux, which was cloned decades ago. The *Dw3* mutant allele contained an 882 bp tandem duplication in exon 5 that abolished the enzyme’s function. This allele is known to be unstable, with the progeny of a homozygous *dw3* plant reverting to a tall *Dw3* wild type at a rate of around 1/600 [10]. In 2024, three InDel mutations in the *Dw3* gene, including an 82 bp deletion, a 6 bp duplication, and a 15 bp deletion, were discovered [11].

Although *Dw4* has not been cloned, it is inferred to be located on chromosome 4 or 6 [12,13]. In the early stages of hybrid utilization in China, sorghum primarily exhibited a “one-dwarf”, reaching heights of 2.5–3 m, leading to serious lodging. Recognizing the need for dwarf hybrids (approximately 2 m in height) for efficient production, Liang et al. advocated for sorghum dwarf breeding [14].

Bulked Sergeant Analysis (BSA) is a fast, convenient, and efficient method of determining the linkage between markers and traits by constructing DNA pools of extreme traits. It has been widely adopted in gene-mapping studies in recent years [15,16,17,18,19]. Compared with traditional linkage mapping, where progeny are individually genotyped and phenotyped, BSA requires the preparation of fewer samples for genotyping and allows for more efficient phenotyping. This is because only individuals that represent the extreme phenotypes of a population need to be identified [15].

China is the world’s eighth-largest producer of sorghum, which is mainly used as food, feed biofuels, and alcoholic beverages. It was introduced through the spreading of Indian cultivars during the silkworm trade or directly from the eastern coast of Africa by returning Chinese seamen [19,20]. Between the 1930s and 1950s, sorghum was grown as a traditional cereal on 10 million hectares, but the production area drastically declined to about 2 million hectares by 1990 and then further to 0.6 million hectares by 2008. Instead, sorghum has become increasingly popular as animal feed, and in recent years, the Chinese government has invested in research into improved varieties for this purpose [3]. In addition, sorghum is also a major ingredient in liquor production in China’s food industry [21]. Almost all famous Chinese brands of distilled liquors, such as Moutaijiu (Moutai aroma liquor), Luzhoulaojiao, Wuliangye (strong aroma liquor), and Fenjiu (light aroma liquor), are brewed with sorghum grain as a key ingredient [22]. Sorghum has been planted in China for a long time and is rich in germplasm resources. Therefore, finding new genes by typing the genes affecting plant height in common sorghum cultivars in China is helpful. The corresponding research is of great benefit to sorghum breeding.

In this study, the genotypes were systematically investigated in 634 sorghum cultivars, which contain a number of Chinese sorghum landraces, and 4 cultivars were identified, *Dw1Dw2Dw3,* with significant differences in plant height. Subsequently, two groups of plants were generated for BSA-seq analysis to map the new genes affecting plant height. By using a recombinant-derived progeny testing strategy [23,24], the candidate gene was narrowed to a region that includes nine genes between 59.75 and 59.86 Mbp. Amino acid sequence analysis of the parent and offspring plants revealed that the novel mutated *Dw3* genes, rather than the novel genes, play a role in reducing the plant height of 8R262 or 8R417. The genotypes of 19 sorghum cultivars primarily exhibited a “one-dwarf” hybrid or wild-type (*Dw1Dw2Dw3*), and a plant height of less than 2 m was identified. Meanwhile, two novel allelic mutations were identified in Chinese sorghum landraces, and another one was inferred through the sequencing results of the Indian cultivars. Consequently, this discovery pointed to the potential existence of different dwarf mutations in the *dw3* gene, some of which could be additional stable sources of the trait. Overall, our study provides new insights into the *Dw3* gene and novel theoretical support for its applications.

## 2. Results

### 2.1. Phenotyping and Genetic Analysis of Dwarf Sorghum Cultivars

In order to seek the fourth or more genes that affect sorghum height, 634 sorghum cultivars were planted in Shenyang city (42° N, 123° E) and numbered to investigate plant height and genotypes. We genotyped *Dw1* and *Dw2* via Sanger sequencing and *Dw3* using the PCR amplification method, which has been commonly employed because the *dw3* mutant allele contains an insertion of a fragment that can be clearly identified [10,25].

As shown in Table 1 and Table S1, the plant heights of three Chinese sorghum landraces (8R262, 8R277, and 8R417) and one sorghum cultivar (8R037) from Morocco were investigated. The plant heights of 8R262 and 8R417 were 107 ± 10 cm and 133 ± 6 cm, while those of 8R277 and 8R037 were 170 ± 10 cm and 175 ± 7 cm, respectively. Although they differed significantly in plant height, all four cultivars were identified as *Dw1Dw2Dw3* and selected for subsequent hybridization manipulations because they had relatively close heading times.

**Figure 1:**
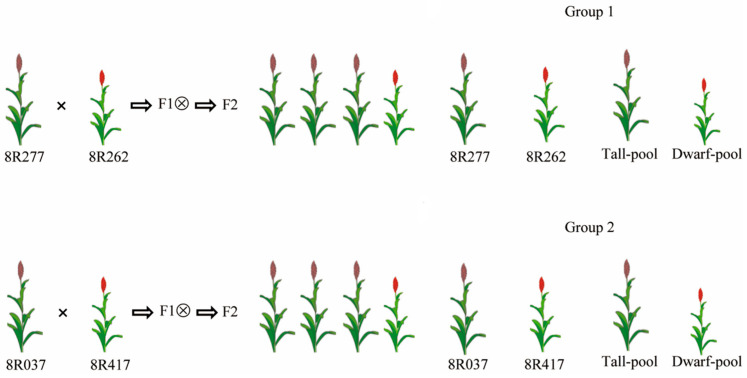
Crossing and extreme pool design.

The tall cultivars, 8R277 and 8R037, were used as the female parents to cross the dwarf cultivars 8R262 and 8R417, respectively, in an inheritance study and during the construction of the mapping population (Table 2, Figure 1). All F1 plants showed a tall plant height, and the F2 plants displayed different heights corresponding to the parents. Therefore, the dwarf-to-tall was a completely recessive trait in two groups of populations. According to the law of segregation, the ratio of dominant-trait individuals to recessive homozygous-trait individuals was 3 to 1 in F2 populations. Among the 708 F2 individuals in the 8R262 × 8R277 population, 536 exhibited a tall plant height, and 172 showed a dwarf plant height, corresponding to a segregation ratio of 3:1 determined using the Chi-square test (χ^2^ = 0.19 < 3.84, *p*-value = 0.67 > 0.05). χ^2^ ≤ 3.84 indicates that the theoretical segregation ratio significantly correlates with the actual ratio. Similarly, in the 8R037 × 8R417 population, F2 individuals also showed a 3:1 segregation ratio of tall and dwarf (χ^2^ = 0.99 < 3.84, *p*-value = 0.32 > 0.05) (Table 2). These results demonstrate that a single recessive gene should control the dwarf trait.

### 2.2. BSA-Seq Analysis and Mapping of Candidate Gene

To map the new genes affecting plant height, we generated two groups of plants for BSA-seq analysis, as shown in Figure 1. Fifty plants with an extreme plant height were selected as the highly tall pool or extreme dwarf pool in both groups, respectively. On the Illumina HiSeq sequencing platform, a total of 725,474,232 raw reads for group 1 and 771,050,148 raw reads for group 2 were generated after sequencing the two groups of parent plants and extreme mixing pools (Table 3). By using quality-control measures, such as removing joints and low-quality reads, 108,821,134,800 and 115,657,522,200 clean bases for groups 1 and 2 were obtained, respectively. The quality of the sequencing data was evaluated, and the sequencing error rate and statistical Q20, Q30, and GC contents were calculated, as shown in Table 2. The quality evaluation showed that the GC contents of these reads were 43.64–43.78% (group 1) and 44.62~45.55% (group 2). The two groups of sequencing data had high quality (Q20 ≥ 93%, Q30 ≥ 97) and could be adopted for further analysis.

**Figure 2:**
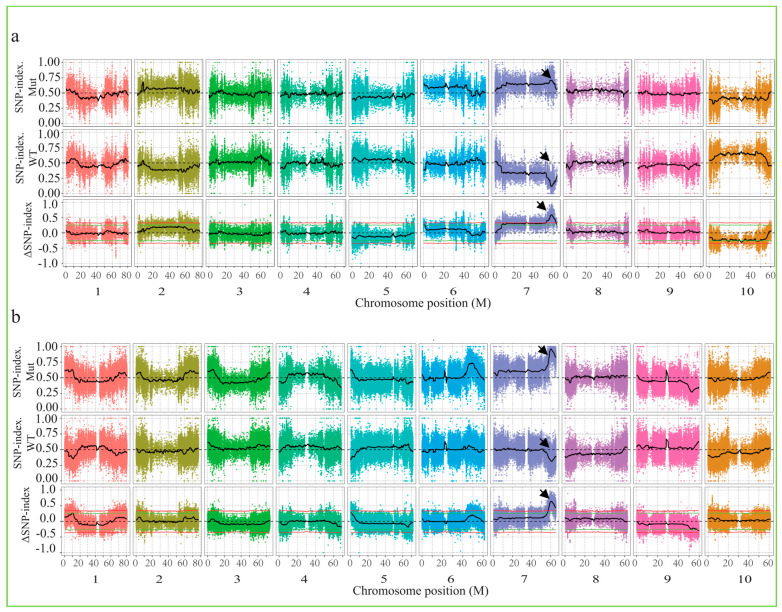
Genome-wide association mapping of plant height. The candidate SNPs and InDels of group 1 (**a**) and group 2 (**b**) using the ΔSNP-index algorithm with a cutoff of ΔSNP-index > 0.5. The arrow indicates the region where the predicted candidate gene was located.

The GATK software toolkit (Version 4.1.8.1) was used for SNP detection and filtration. A total of 862,470 (group 1) and 1,285,629 (group 2) SNPs were identified, of which 45,340 were located in the exon regions, including 29,271 non-synonymous mutations, 545 stop gain mutations, and 75 stop loss mutations (group 1). The data from group 2 suggest that 67,802 were located in the exon regions, containing 47,584 non-synonymous mutations, 847 stop gain mutations, and 133 stop loss mutations. To determine the genomic region linked to plant height, we utilized the ΔSNP-index method to assess the allele segregation of the SNPs and InDels between the two DNA pools representing the extreme groups. According to the fitted △SNP-index computer simulation experiment, taking the threshold of 99% confidence (red line in the figure), the association region was located in the range of 10.5 Mb on chromosome 7 of 8R262 plants. Meanwhile, it was located within an 8.2 Mb region on chromosome 7 of 8R417 plants, as indicated by the arrows in Figure 2.

**Figure 3:**
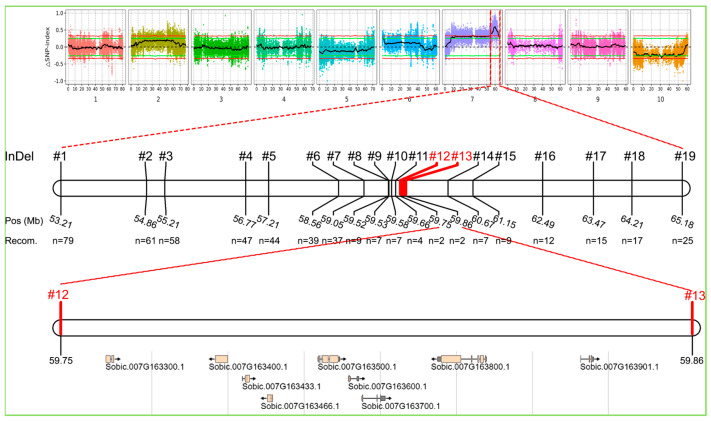
Fine mapping of the candidate gene. Narrowing the candidate region through recombinant-derived progeny testing strategy produced the genetic linkage map of a candidate region of Chr.7. The genes and diagnostic markers in the 0.11 Mb interval.

After several screenings, we successively obtained 123 recombinants and narrowed the candidate gene to an interval flanked by the markers InDel-6 and InDel-15. Coincidentally, the interval almost coincided with the region of the most credible prediction in BSA-seq analysis. To further locate the candidate gene, another 10 InDel markers were applied to seek the limited individuals’ recombinants. Due to the finding of the last four recombinants, the candidate gene was narrowed to a region that includes nine genes between 59.75 and 59.86 Mbp (Figure 3). *Dw3* was among them, as annotated and listed in Table 4. Moreover, a similar experiment was carried out in group 2, and consistent results were obtained.

### 2.3. Identification of the Candidate Gene Related to Plant Height in Sorghum Plants

**Figure 4:**
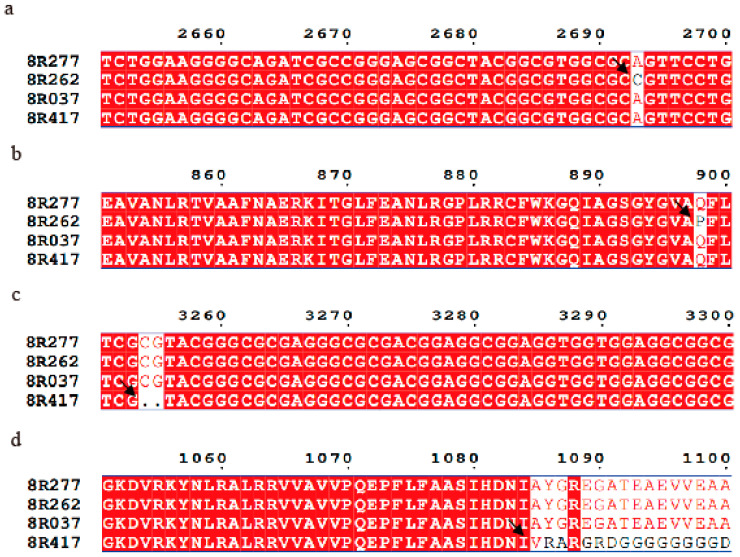
CDS and amino acid sequence analysis of *Dw3* genes from different cultivars. The CDS (**a**,**c**) and amino acid (**b**,**d**) sequences of Dw3 were arranged to show the variations, respectively. Multiple sequence alignment was performed using the espript 3.0 online tool (https://espript.ibcp.fr/ESPript/cgi-bin/ESPript.cgi (accessed on 1 September 2024)). Arrows point to locations where there are variations in the sequences.

We performed Sanger sequencing for all nine genes in the male and female parents to identify the candidate gene. Although the DNA sequences of the nine genes were not identical, only Dw3 had a non-synonymous mutation in the amino acid sequence of 8R262 or 8R417 plants, as shown in Figure 4. To illustrate the variations, CDS sequences (Figure 4a,c) and amino acid sequences (Figure 4b,d) of Dw3 were arranged for further analysis. In contrast to 8R277 and other cultivars, the amino acid sequence of 8R262 had a substitution of proline for glutamine at position 898 (Figure 4b), which was caused by the conversion of “A” to “C” at position 2693 in the CDS sequence (Figure 4a). The CDS sequence 8R417 also had a two-base deletion at position 3254 (Figure 4c), resulting in the frameshift mutation starting at position 1085 (Figure 4d). 

Furthermore, we identified the *Dw3* gene of 8R262 and 8R417 varieties again using the traditional PCR method, and the results showed that the *Dw3* gene of these two varieties did not have fragment insertion compared with the BTx623 sorghum reference genome (Figure S1). The integrated results revealed that the novel mutated *Dw3* genes, rather than a novel gene, play a role in determining the plant height of 8R262 and 8R417. Hence, the genotypes of some common sorghum cultivars primarily exhibiting a “one-dwarf” hybrid or wild-type (*Dw1Dw2Dw3*) and plant heights of less than 2 m are re-summarized and listed in Table 5 since two novel mutant forms of *Dw3* were identified. Furthermore, the *Dw3* genes of these cultivars were also determined using Sanger sequencing. As shown in Table S2, both the CDS sequences of 8R258 and 8R428 also had a two-base deletion at position 3254, consistent with 8R417, and a significantly lower plant height. Therefore, it can be inferred that this type of mutation is not an isolated phenomenon. In addition, in contrast to other cultivars, the amino acid sequences of 8R019, 8R100, and 8R402 had a substitution of arginine for lysine at position 985, which was caused by the conversion of “A” to “G” at position 2954 in the CDS sequence (Table S2). At the same time, the plant height of the cultivars decreased significantly, which may be a new mutation type and needs further determination.

## 3. Discussion

Over the past few years, the application of BSA-seq technology in sorghum gene mapping has gradually attracted the attention of researchers. In 2022, Mendu et al. identified and characterized three allelic thick leaf mutants (*thl1*, *thl2*, and *thl3*). BSA-seq analysis showed that the causal mutation for the *thl* phenotype is in the endo-1,4-β-glucanase gene (*SbKOR1*) [26]. De Riseis et al. identified two distinct mutations in the Sorghum bicolor *Id1* (*SbId1*) homolog through BSA-seq analysis, which both delayed the flowering time [27]. Moreover, Demeke B. Mewa reported mapping via BSA-seq of ANTHRACNOSE RESISTANCE GENE 2 (ARG2) encoding a nucleotide-binding leucine-rich repeat (NLR) protein that confers race-specific resistance to *Colletotrichum sublineola* strains [28]. These successful examples indicate the feasibility of using BSA-seq in this study to map genes affecting the height of sorghum plants.

A great many factors can affect plant height. In sorghum, plant height is controlled by a few major dwarfing genes. Quinby and Karper identified four loci (*Dw1*–*Dw4*) that control the internode length by measuring the height of the stem from the ground to the flag leaf. At each Dw locus, the dominant allele increased the internode length [5,29]. Only three of the four genes (*Dw1*–*3*) were mapped and cloned, and they were located on chromosomes 9, 6, and 7, respectively [6,8,10].

The fourth classical dwarfing locus in sorghum, *Dw4*, is known to be unlinked to the other dwarfing loci and is believed to be on chromosome 4 or 6, but associated genes remain uncloned [12,13]. In our study, 19 markers were developed after BSA-seq analysis. After several rounds of screening, the gene affecting plant height was mapped on chromosome 7 (Figure 2), which was quite different from previous inferences for *Dw4* [12].

Since the nonfunctional *dw3* allele is the result of an 882 bp tandem duplication in exon 5 that disrupts protein function and the plant’s ability to establish an auxin gradient in the intermediate internodes, the PCR amplification method was adopted to identify mutations [10]. As illustrated in Table 1, although no fragment insertion was observed in the *Dw3* gene sequences, plant height was lower in several cultivars. Subsequently, in the BSA-seq analysis, genes affecting plant height were mapped to an interval containing nine open reading frames (ORFs). At the same time, the Sanger sequencing results showed that only the *Dw3* gene had a non-synonymous mutation in the amino acid sequence (Figure 4). The mutant form of the *Dw3* gene differed from previous studies [10,25]. This allele is known to be unstable, with the progeny of a homozygous *dw3* plant reverting to a tall *Dw3* wild type at a rate of around 1/600 [10].

All the results above suggest that mutations in the *Dw3* gene reduce plant height, and two novel mutation forms were identified.

In China, sorghum has been a critical ingredient for brewing liquors since the Yuan Dynasty (1270-1368 CE) [30,31]. Sorghum is mainly cultivated in China’s southwest, north, and northeast regions, and more than 80% of sorghum grain is used for liquor-making [18,32]. Therefore, there are abundant sorghum germplasm resources in China, which provide more convenience for the selection and breeding of new cultivars. This study systematically identified the main genotypes affecting plant height genes of 19 sorghum cultivars (Table 5), which contain a number of Chinese sorghum landraces. In our study, two novel allelic mutations identified using BSA-seq were only found in Chinese sorghum landraces. In addition, we also inferred another allelic mutation by studying the sequencing results of the Indian cultivars (8R019 and 8R100) or 8R402 (a Chinese sorghum landrace). Our article pointed to the potential existence of different dwarf mutations in the *dw3* gene, some of which could be additional stable sources of the trait.

## 4. Materials and Methods

### 4.1. Plant Materials, Phenotyping, DNA Extraction, and Sanger Sequencing

To investigate plant height and genotypes, 397 sorghum cultivars were obtained from the China National Center for Sorghum Improvement. Similarly, 237 sorghum cultivars were obtained from the Sorghum Mini Core Collection (MCC) to identify additional genes influencing sorghum height [33]. The field trials to evaluate the phenotypic performance of sorghums were conducted in Shenyang city (42° N, 123° E) in the 2014 summer season.

In order to improve pollination success and find suitable crosses, the heading date (HD) was recorded when 50% of the plants in the plot showed 50% flowering. Three plots of sorghum plants of each cultivar were planted. Three individual plants from the middle of each plot were selected for phenotype evaluation. Plant height was evaluated at maturity, as described by Zou et al. [34].

Genomic DNA (5–6 plants per accession) was extracted from 30-day-old seedlings by using the CTAB protocol [35]. DNA concentration and quality were estimated with an ND-1000 spectrophotometer (NanoDrop, Wilmington, DE, USA) and by electrophoresis on 1.0% agarose gels with a lambda DNA standard.

Fragments of *Dw1*, *Dw2,* and *Dw3* were amplified. PCR was performed in a 25 μL reaction containing 2× EXtaqMix (Takara, Kusatsu-shi, Japan), 0.4 μM of each primer, 0.125 μg of DNA, and double-distilled water. A PCR protocol was used to amplify the fragments, including an initial denaturation cycle at 95 °C for 4 min, followed by 30 cycles of denaturation at 95 °C for 30 s, annealing at 57 °C for 30 s, and extension at 72 °C for 2.5 min. Primers for Sanger sequencing and PCR-amplification were listed in Table S3, and PCR products were sequenced by Biomarker Technologies Corporation (Beijing, China).

### 4.2. SNP Library Construction and High-Throughput Sequencing

The specific length amplified fragment sequencing (SLAF-seq) employed in this study was performed following Sun et al. with minor modifications [36]. The SLAF library was constructed as follows. First, a SLAF pre-design experiment was performed to establish conditions to optimize SLAF yield, avoid repetitive SLAFs, and obtain an even distribution of SLAFs for maximum SLAF-seq efficiency. These considerations improved the efficiency of SLAF-seq. Next, the SLAF library was constructed in accordance with the pre-designed scheme. Genomic DNA was incubated at 37 °C with *Mse* I (New England Biolabs, NEB.; Ipswich, MA, USA), T4 DNA ligase (NEB), ATP (NEB), and *Mse* I adapter. The reaction was heat-inactivated at 65 °C and then digested for the additional restriction enzyme *Alu* I at 37 °C. Then, polymerase chain reactions (PCRs) were carried out using diluted restriction ligation samples, dNTP, Taq DNA polymerase (NEB), and *Mse* I primer containing barcode1. Using E.Z.N.A.H Cycle Pure Kit (Omega, Biel/Bienne, Switzerland), the PCR productions were purified and pooled. The pooled samples were incubated at 37 °C with *Mse* I, T4 DNA ligase, ATP, and Solexa adapter, purified using a Quick Spin column (Qiagen, Hilden, Germany), and then run out on a 2% agarose gel. Using a Gel Extraction Kit (Qiagen, Hilden, Germany), fragments of 450–500 bp (with indexes and adaptors) in size were isolated. These fragment productions were then subjected to PCR amplification with Phusion Master Mix (NEB) and Solexa Amplification primer mix (Illumina, Inc., San Diego, CA, USA) to add barcode 2 according to the Illumina sample preparation guide.

Samples were gel-purified by excising DNA of 450–500 bp and diluted for paired-end sequencing on an Illumina HiSeq 2000 sequencing platform (Illumina, Inc., San Diego, CA, USA) in Beijing (http://www.biomarker.com.cn (accessed on 7 November 2024)). SNP genotyping and evaluation were then performed.

All SLAF paired-end reads with clear index information were clustered according to sequence similarity, which was detected using one-to-one alignment by BLAT (−tileSize = 10 − stepSize = 5) [37]. Sequences with over 90% identity were grouped in one SLAF locus. Using the minor allele frequency (MAF) evaluation, alleles were defined in each SLAF.

In mapping populations of sorghum, one locus can contain at most four genotypes, so the groups containing more than four tags were filtered out as repetitive SLAFs. SLAFs with a sequence depth of less than 107 were filtered out of the following analysis. SLAFs with 2–4 tags were identified as polymorphic SLAFs, which were considered potential markers. Tags with at least 10× total coverage were retained. Sequences were mapped to the BTx623 sorghum reference genome using BWA [38,39], and SNPs were called with the TASSEL 3.0 GBS pipeline (www.maizegenetics.net/tassel/ (accessed on9 January 2024)). Missing data were imputed with NPUTE [40].

## 5. Conclusions

Three novel allelic mutations were discovered by BSA-seq analysis and amino acid sequence alignment. Two of them were only identified from Chinese sorghum landraces. Multiple types of allelic mutations in the *Dw3* gene should be considered when identified or applied.

## Figures and Tables

**Table 1:** Genotyping of genes affecting plant height and statistics of plant height and heading date.

Cultivar Number	Plant Height (cm)	Heading Date (Day)
8R277	170 ± 10 a	68
8R262	107 ± 10 c	65
8R037	175 ± 7 a	72
8R417	133 ± 6 b	70

Data marked with different letters (a–c) indicate significant differences, as analyzed by SPSS 23.0 software (Duncan’s multiple range test, α = 0.05).

**Table 2:** The phenotype analysis results of tall/dwarf plant height in the two sorghum populations.

Population	Generation	Total Plants	Tall	Dwarf	^a^ Expected Ratio	^b^ χ^2^	^c^ *p*-Value
8R262 × 8R277	F_2_	708	536	172	3:1	0.19	0.67
8R037 × 8R417	F_2_	594	456	138	3:1	0.99	0.32

^a^ Expected ratio: dominant-trait individual: recessive-trait individual = 3:1. ^b^ χ^2^: χ^2^ < 3.84 is considered significantly correlated. ^c^ *p*-value: *p*-value > 0.05 indicates no statistically significant difference.

**Table 3:** Sequencing statistics for BSA-seq samples.

	Sample	Clean Reads	Clean Bases	Mapped (%)	Properly Mapped (%)	Average Depth	Q20 (%)	Q30 (%)	GC Content (%)
Group 1	B8R262	106,444,214	15,966,632,100	98.40	90.03	21	97.71	93.38	43.64
	B8R277	110,755,784	16,613,367,600	98.33	89.73	22	97.80	93.62	43.78
	Dwarf pool	256,010,212	38,401,531,800	96.04	89.16	49	98.18	94.43	44.63
	Tall pool	252,264,022	37,839,603,300	97.81	90.06	49	97.88	93.66	43.71
Group 2	B8R037	59,041,644	8,856,246,600	96.15	88.82	11	98.09	94.15	45.21
	B8R417	62,894,480	9,434,172,000	96.94	90.73	12	97.71	93.37	45.55
	Dwarf pool	338,573,164	50,785,974,600	94.91	87.23	62	98.29	94.77	44.81
	Tall pool	310,540,860	46,581,129,000	96.38	89.46	57	98.31	94.86	44.62

**Table 4:** Gene descriptions from the NCBI of 9 genes in the predictive region.

Gene Number	Annotation
SORBI_3007G163300	NA
SORBI_3007G143400	Tetraspanin-8, *Arabidopsis thaliana* (Mouse-ear cress).
SORBI_3007G163433	NA
SORBI_3007G163466	NA
SORBI_3007G143500	Uncharacterized protein, *Arabidopsis thaliana* (Mouse-ear cress).
SORBI_3007G153600	Protein HVA22, *Hordeum vulgare* (Barley).
SORBI_3007G163700	NA
SORBI_3007G163800	ABC transporter B family member 1 (*Dw3*, Sorghum)
SORBI_3007G163901	NA

Genes were blasted on the NCBI website (https://www.ncbi.nlm.nih.gov/ (accessed on 4 December 2023)). “NA” indicates no annotations.

**Table 5:** The genotyping of genes affecting plant height.

Cultivar Number	Origin	Genotype	Plant Height (cm)
8R019	India	*Dw1dw2dw3*	111 ± 16 ef
8R037	Morocco	*Dw1Dw2Dw3*	170 ± 10 abc
8R082	South Africa	*Dw1Dw2Dw3*	192 ± 11 ab
8R100	India	*Dw1dw2bdw3*	122 ± 12 ef
8R105	Sultan	*dw1Dw2Dw3*	121 ± 21 ef
8R151	India	*Dw1Dw2Dw3*	199 ± 48 a
8R205	Zimbabwe	*Dw1dw2Dw3*	142 ± 11 cde
8R206	Zimbabwe	*Dw1dw2Dw3*	144 ± 8 cdef
8R219	South Korea	*Dw1dw2Dw3*	165 ± 7 abcd
8R260	China	*Dw1Dw2Dw3*	184 ± 17 ab
8R258	China	*Dw1dw2Dw3*	129 ± 6 def
8R262	China	*Dw1Dw2dw3*	107 ± 10 f
8R277	China	*Dw1Dw2Dw3*	169 ± 10 abc
8R281	China	*Dw1Dw2Dw3*	160 ± 24 bcd
8R315	India	*Dw1dw2Dw3*	157 ± 22 bcd
8R390	The United States	*Dw1dw2Dw3*	159 ± 11 bcd
8R402	China	*Dw1Dw2dw3*	122 ± 11 ef
8R417	China	*Dw1Dw2dw3*	120 ± 29 ef
8R428	China	*Dw1Dw2Dw3*	170 ± 4 abc

The genotypes of some common sorghum cultivars primarily exhibiting a “one-dwarf” hybrid or wild-type (*Dw1Dw2Dw3*) and a plant height of less than 2 m are listed in the table. Data marked with different letters (a–f) indicate significant differences, as analyzed using SPSS 23.0 software (Duncan’s multiple range test, α = 0.05).

## Data Availability

The data presented in this study are available upon request from P.W.

## Supplementary Materials

The following supporting information can be downloaded at: https://www.mdpi.com/article/10.3390/ijms252212000/s1.
