# Supplementary figures and images for: Novel Allelic Mutations in Dw3 Gene That Affect the Height of Sorghum Plants
Source: Int J Mol Sci. 2024 Nov 8;25(22):12000. doi: 10.3390/ijms252212000 (PMC11593585; doi:10.3390/ijms252212000)

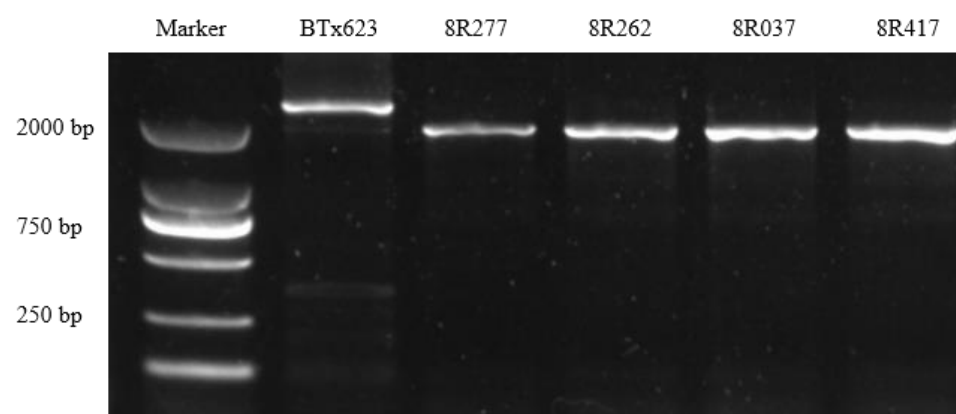

Figure S1 PCR identification of *Dw3* gene

Supplement: Supplementary file 1 [file ijms-25-12000-s001.zip › Figure S1.pdf]
